# Choroidal Neovascularization Is Inhibited in Splenic-Denervated or Splenectomized Mice with a Concomitant Decrease in Intraocular Macrophage

**DOI:** 10.1371/journal.pone.0160985

**Published:** 2016-08-17

**Authors:** Xue Tan, Katsuhito Fujiu, Ichiro Manabe, Junko Nishida, Reiko Yamagishi, Yuya Terashima, Kouji Matsushima, Toshikatsu Kaburaki, Ryozo Nagai, Yasuo Yanagi

**Affiliations:** 1 Department of Ophthalmology, Graduate School of Medicine and Faculty of Medicine, The University of Tokyo, Tokyo, Japan; 2 Department of Cardiovascular Medicine, Graduate School of Medicine and Faculty of Medicine, The University of Tokyo, Tokyo, Japan; 3 Department of Ubiquitous Health Informatics, School of Medicine, The University of Tokyo, Tokyo, Japan; 4 Precursory Research for Embryonic Science and Technology, Japan Science and Technology Agency, Tokyo, Japan; 5 Department of Aging Research, Chiba University Graduate School of Medicine, Chiba-shi, Chiba, Japan; 6 Department of Molecular Preventive Medicine, Graduate School of Medicine, The University of Tokyo, Tokyo, Japan; 7 Jichi Medical University, Tochigi, Japan; 8 Singapore Eye Research Institute, Singapore, Singapore; 9 Medical Retina Department, Singapore National Eye Centre, Singapore, Singapore; 10 Duke-NUS (National University of Singapore) Graduate Medical School, Singapore, Singapore; Tohoku University, JAPAN

## Abstract

**Purpose:**

To determine the involvement of sympathetic activity in choroidal neovascularization (CNV) using laser-induced CNV in a mouse model.

**Methods:**

We investigated changes in the proportions of intraocular lymphocytes, granulocytes, and three macrophage subtypes (Ly6C^hi^, Ly6C^int^, and Ly6C^lo^) after laser injury in mice using flow cytometry, and evaluated CNV lesion size in mice lacking inflammatory cells. Further, we evaluated the lesion size in mice administered the β3 receptor antagonist, splenic-denervated and splenectomized mice. We also assessed changes in the proportions of intraocular macrophages and peripheral blood monocytes in splenic-denervated and splenectomized mice. Lastly, lesion size was compared between splenic-denervated mice with or without adoptive transfer of macrophages following laser injury. After Ly5.1 mice spleen-derived Ly6C^hi^ cells were transferred into Ly5.2 mice, the proportions of intraocular Ly5.1^+^Ly6C^hi^ cells were compared.

**Results:**

In WT mice, the proportion of CD4^+^ T cells recruited into the eye increased progressively from day 3 to day 7 after laser injury, whereas, intraocular CD8^+^ T cells did not change significantly. Proportions of B220^+^ cells, granulocytes, and two subtypes of intraocular macrophages (Ly6C^hi^ and Ly6C^lo^) peaked at day 3 following laser injury. In contrast, Ly6C^int/lo^CD64^+^ subtype showed a significantly higher percentage at day 7 after laser injury. There were no differences in lesion size between *CD4*^*–/–*^or *Rag2*^*–/–*^mice and controls, whereas lesion size was significantly reduced in *CCR2*^*−/−*^ mice and clodronate liposome-treated mice. CNV lesion area was significantly reduced in mice with β3 blocker treatment, splenic-denervated and splenectomized mice compared with controls. Intraocular Ly6C^hi^ macrophages were also reduced by splenic denervation or splenectomy. Adoptive transfer of spleen-derived Ly6C^hi^ cells increased the lesion size in splenic-denervated mice. Compared with controls, intraocular donor-derived Ly6C^hi^ cells recruited into the eye were reduced in splenic-denervated and splenectomized mice.

**Conclusions:**

Although lymphocytes had little effect on CNV formation, Ly6C^hi^ macrophages/monocytes exacerbated CNV in mice. Sympathetic activity might contribute to CNV via the recruitment of macrophages to the eye.

## Introduction

In recent studies, inflammation has been found to play an important role in the development of choroidal neovascularization (CNV) [1] in both experimental and clinical settings. For example, in experimental models, granulocyte infiltration was found to promote laser-induced CNV [2]. Similarly, a histological study of CNV revealed the involvement of macrophages [3], while a depletion of macrophage was found to correlate with reduced CNV formation [4,5]. Conversely, there are reports that intraocular injection of macrophages actually reduces CNV size [6]. These findings suggest that the role of macrophages in CNV is critical and complex and may depend on the age of experimental animals and the age and subtypes of macrophages. Furthermore, the influence of other inflammatory cells besides macrophages on AMD is still controversial. A few studies have also suggested that T cells contribute to CNV formation. Immunization with an age-related macular degeneration (AMD)-specific epitope, carboxyethylpyrrole (CEP), resulted in the production of interferon-gamma (IFN-γ) and interleukin-17 (IL-17)-producing T cells, which then promoted macrophage polarization leading to CNV [7]. On the other hand, Tsutsumi-Miyahara et al found that T cells had little effect on CNV formation in a laser-induced model [8]. In addition, B cells were not detected among ocular infiltrating cells after laser injury [8]. Thus, the precise contributions of specific lymphocyte subtypes to the regulation of angiogenesis in the eye remain unclear.

Mouse blood monocytes are suggested to subdivide into three subtypes, that is, classical, intermediate, and nonclassical. The classical monocytes show high expression of Ly6C, the chemokine receptor CCR2, and only moderate amounts of CX_3_C-chemokine receptor 1 (CX_3_CR1) together with low CD43. These cells have been demonstrated to be rapidly recruited to inflamed tissues, and so Ly6C^hi^ subtype is known as “inflammatory” monocytes. Recent studies have demonstrated that inflammatory monocyte recruitment into the choroid is suppressed by TNFα-stimulated gene/protein (TSG)-6, which is a multifunctional endogenous protein, resulting in reduced CNV lesion size in a rat model of laser-induced CNV [9]. Although a previous report demonstrated that lack of CCR2/CCL2 led to spontaneous development of CNV in mice [10], later studies refuted this by demonstrating that *Rd8* gene mutation, not the lack of CCR/CCL2, was the primary cause of CNV. It is now widely accepted that CCR2 deficiency suppresses CNV in mice without the *Rd8* mutation [11,12]. By contrast, the nonclassical monocytes are considered as the “resident” subset because they were found to be recruited in both resting and inflammatory lesions and persist for longer. They are characterized by high CX_3_CR1 expression, low expression of Ly6C and CCR2 together with high CD43. The Ly6C^lo^ subtype has been demonstrated to be differentiated from Ly6C^hi^ subtype in the peripheral blood [13]; however, recent studies have suggested tissue-resident macrophages originate from the yolk sac or fetal liver cells and subsequently maintaining themselves through longevity and limited self renewal [14,15]. The intermediate monocytes were identified as a rare population of monocytes that were characterized by an intermediate level of Ly6C and high CD43 [16]. The Ly6C^int^ subset might be an intermediate function state adopted by Ly6C^hi^ cells before they turn into the Ly6C^lo^ subtype [17]. Three monocyte subtypes can also be identified in humans with markers CD14 and CD16, and show similar phenotypes to those of mice [16]. Although it may be difficult to differentiate between the subtypes due to conversion from classical to nonclassical monocytes; inflammatory and resident monocytes appear to be two distinct subpopulations with different characters and functions.

Bone marrow and peripheral blood are the primary sources of infiltrating monocytes after injury [18]. Pain, anxiety and tissue injury can heighten sympathetic nervous system activity, and sympathetic nervous system activity can release hematopoietic stem cells from bone marrow via the β3 adrenergic receptor [19]. Recently, the spleen was demonstrated to function as a reservoir for monocytes. After ischemic myocardial injury, splenic monocytes migrated to injured tissue to regulate inflammation. Treatment with a β3 adrenergic blocker also reduced splenic accumulation of progenitors in wild type mice after myocardial infarction [20]. A previous study demonstrated that choroidal thickness and ocular blood vessel number were increased by long-term cervical sympathectomy [21]. However, there are few studies on the link between sympathetic nervous system and CNV.

As a first step to directly examine the role of inflammatory cells in the development of CNV, we used flow cytometry to evaluate temporal changes in the proportions of lymphocytes, granulocytes, and macrophages in the posterior segment of the eye. Then, we used a laser-induced CNV model to examine lesion size in mice lacking inflammatory cells or macrophage-depleted mice. Furthermore, to investigate the involvement of sympathetic activity in ocular angiogenesis, we examined changes in CNV after splenic denervation or sympathetic nerve blockage by β3 receptor antagonist treatment in laser-induced CNV mice, together with the concomitant change in the proportion of intraocular macrophage subtypes using flow cytometry. Lastly, we transferred Ly6C^+^ cells sorted from spleen into splenic-denervated mice before laser photocoagulation to examine the involvement of Ly6C^+^ macrophages/monocytes in the process of CNV formation. We also transferred Ly6C^+^ cells sorted from the spleen of Ly5.1 mice to Ly5.2 mice, and evaluated the proportion of spleen-derived Ly6C^hi^ cells that were recruited into the eye.

## Materials and Methods

All procedures were performed in accordance with the ethical guidelines for animal experimentation of the University of Tokyo and the Association for Research in Vision and Ophthalmology. The study was approved by the Institutional Animal Research Committee of the University of Tokyo.

### Animals

Male wild-type (WT) C57BL/6J mice were purchased from Kiwa Laboratory (Wakayama, Japan). Male CD4-deficient (*CD4*^*−/−*^) mice, male Rag2 deficient (*Rag2*^*–/–*^) mice, which lack mature T and B lymphocytes due to an inability to initiate rearrangement of the T cell receptor and Ig loci, and Male C-C chemokine receptor type 2 (CCR2)-deficient mice (*CCR2*^*−/−*^) were purchased from Taconic Biosciences (Germantown, NY). All mice were used at 7 to 8 weeks of age. Genotyping the mice for the *Rd8* mutation was performed as described previously [22]. Anesthesia was achieved by intraperitoneal injection of 75 mg/kg ketamine HCL (Ketalar^®^; Sankyo, Tokyo, Japan) and 5 mg/kg xylazine (Celectal^®^; Bayer, Tokyo, Japan). Pupils were dilated with a solution of 0.5% tropicamide (Mydrin-M^®^; Santen, Osaka, Japan).

### Laser-induced CNV

CNV lesions were induced by laser photocoagulation as previously described [23–27] using a diode laser (DC-3000^®^; NIDEK, Osaka, Japan) and a slit lamp delivery system (SL-7F; Topcon, Tokyo, Japan) with a cover slip as a contact lens. Laser photocoagulation (200 mW intensity, 170 μm size, 0.02 s duration) was applied to 4 spots per eye for the lesion size studies and to 12 spots per eye for the flow cytometric studies. Laser irradiation was delivered between the major retinal vessels, 2 to 3 disk diameters from the optic nerve. Bubble formation at the time of photocoagulation was used as an indication of Bruch's membrane rupture. In order to minimize the effects of subjective bias when allocating animals to treatment, a randomization procedure was used. Each treatment group consisted of 5 to 8 mice. For the comparative studies of CNV area, 6 or 8 mice per group were used for preparing the RPE flatmounts. For flow cytometric analysis, cells isolated from 5 to 6 mice were used for a single FACS analysis, and each experiment was performed in triplicate. Thus, a total of 309 wild-type mice, 8 *CD4*^*−/−*^ mice, 8 *Rag2*^*–/–*^mice and 53 *CCR2*^*−/−*^ mice were used for the experiments.

### RPE flatmounts

One week after laser injury, mice were perfused transcardially with PBS and then with FITC-conjugated concanavalin A (20 μg/mL in PBS; Vector Laboratories, Burlingame, CA) to label the CNV lesions. The eyes were harvested and the RPE/choroids prepared as flatmounts. CNV lesions in the choroidal flatmounts were observed using a fluorescence microscope (Keyence Corporation, Tokyo, Japan). Lesion area was measured by using Image J software (developed by Wayne Rasband, National Institutes of Health, Bethesda, MD; available at http://rsbweb.nih.gov/ij/index.html) in a masked manner. The outline of the CNV was drawn around the perimeter of the lesion and then the total lesion area was measured. Mean CNV area obtained from the 4 lesions in each eye was used to give a single value for analysis. Each treatment group consisted of 6 or 8 mice.

### Flow cytometry

Peripheral blood was collected from the heart with 1000 units/mL heparin (Novo-Heparin^®^; Mochida, Tokyo, Japan). After erythrocytes were lysed and removed by BD PharmLyse (BD), peripheral blood samples were prepared for flow cytometric analyses [28]. Retina and RPE/choroid were harvested from 10 or 12 eyes of each group and mixed with collagenase (1 mg/mL; Wako, Osaka, Japan) and dispase (1 mg/mL; Invitrogen) in PBS. After 8 min of incubation at 37°C, cells were passed through 40-μm nylon mesh and then centrifuged at 900 × g for 10 min at 4°C. Single-cell suspensions were subjected to flow cytometry. All analyses were performed using a FACSAria III system (BD Biosciences) with FlowJo software (Tree Star). Anti-CD3-PE-Cy7, anti-CD4-PE, anti-CD8-APC, anti-B220-FITC, anti-F4/80-APC, anti-Ly6G-APC-Cy7, anti-CD45.2-FITC, anti-CD115-PE, anti-Ly6C-Pacific Blue, anti-CD64-PE, anti-CD45.1-APC (all from BioLegend), anti-CD11b-PE/Cy7, and anti-CD43-APC antibody (BD Pharmingen) were used for labeling/cell sorting. Lymphocytes (T cells and B cells) were identified as CD3^+^CD4^+^/CD8^+^ or B220^+^, granulocytes as CD11b^+^F4/80^-^Ly6G^+^. To identify macrophage/monocyte subpopulations, blood samples were subjected to staining for CD11b, Ly6G, F4/80, Ly6C, CD45, CD115, CD43, and eye samples were stained for CD11b, Ly6G, F4/80, Ly6C, CD45 and CD64. Using gating strategies as previously described [28,29], we could identify three subpopulations (Ly6C^hi^, Ly6C^int^ and Ly6C^lo^) more clearly using Ly6C and CD43 for peripheral blood monocytes, than using Ly6C and F4/80 (S1 Fig). In contrast, by using Ly6C and CD64 for intraocular macrophages [29], we could identify three relatively clear ocular subpopulations (Ly6C^hi^, Ly6C^lo^ and Ly6C^int/lo^CD64^+^) (S2 Fig).

### Treatment with clodronate liposomes

Splenic and systemic macrophage depletion in C57BL/6J mice (n = 6) was achieved by intraperitoneal administration (i.p.) of 100 μL/10 g body weight of clodronate liposomes (FormuMax Scientific Inc, Palo Alto, CA) 24 h before laser photocoagulation and 50 μL/10g body weight 4 days after laser injury. Control mice were injected with control liposome vehicle (FormuMax Scientific Inc, Palo Alto, CA).

### Treatment with β3 receptor antagonist

WT mice were injected i.p. with SR59230A (Sigma-Aldrich), a selective antagonist for the adrenergic β3 receptor [30,31], twice daily at 5 mg/kg body weight in 100 μL PBS. Mice injected with equal volume PBS were used as a control [32]. Each treatment group consisted of 6 mice.

### Denervation of the spleen

The splenic bundle (consisting of the splenic artery, splenic vein, and splenic nerve) was carefully separated from the surrounding connective tissue under a microscope. The splenic nerve was then detached from the splenic vessels and discarded. Sham-operated mice were used as a control. 6 mice per group were used for the analysis of CNV area, and cells isolated from 5 mice were used for a single FACS analysis.

### Splenectomy

After the spleen was identified, the splenic arteries and venous supply were carefully ligated, thus effectively removing the spleen. Sham-operated mice were used as a control. 6 mice per group were used for the analysis of CNV area, and cells isolated from 5 mice were used for a single FACS analysis.

### Adoptive transfer of splenic monocytes/macrophages

First, CD11b^+^CD115^+^Ly6C^hi^ cells were isolated from spleen by fluorescence cell sorting (FACSAria III, BD Biosciences), resuspended in sterile PBS at 6 × 10^5^ cells/ml, and injected (1.2 × 10^5^ in 200 μL per animal) into the tail vein of mice before laser injury. Laser irradiation was delivered to each eye (4 spots/eye) at the same day, and seven days later CNV lesion size was evaluated by RPE/choroid flatmounts. Finally, CD11b^+^CD115^+^Ly6C^hi^ cells, which were sorted from the spleens of Ly5.1 mice (n = 4) by fluorescence cell sorting (FACSAria III, BD Biosciences), were injected into the tail vein of controls, splenic-denervated and splenectomized mice, which were all prepared from Ly5.2 mice (3 × 10^4^ cells in 200 μL per animal, n = 5). At the same day, laser irradiation was delivered to each eye (12 spots/eye) and three days later, the proportion of intraocular Ly5.1 mice-derived Ly6C^hi^ cells was measured using flow cytometric study.

### Statistical Analysis

Statistical significance was analyzed using the Wilcoxon rank-sum test or the Dunn's multiple comparison test for post-hoc analysis using JMP Pro 10 software (SAS). P values less than 0.05 were considered statistically significant.

## Results

All strains of transgenic mice and their wild-type littermates were Rd8 negative and had similar retinal morphology without laser treatment.

### Ocular infiltration of immune cells after photocoagulation

**Fig 1:**
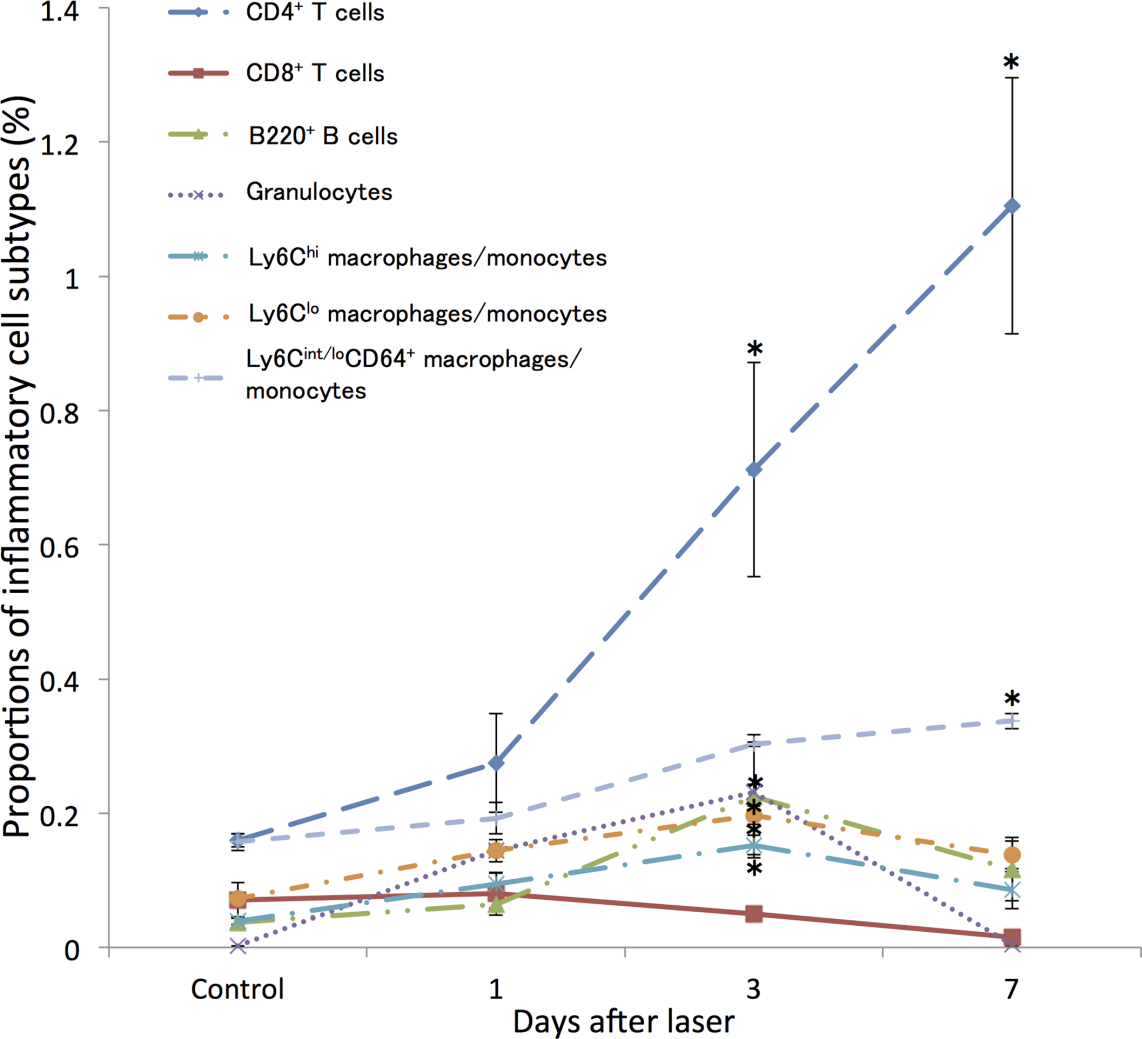
Change in the proportions of inflammatory cells recruited into the posterior segment of the eye in wild-type mice following laser photocoagulation. The proportion of CD4^+^ T cells increased progressively from day 3 after laser injury and greatly increased until day 7. Proportions of B220^+^ cells, granulocytes, and two subtypes of intraocular macrophages (Ly6C^hi^ and Ly6C^lo^) peaked at day 3 after laser injury, whereas Ly6C^int/lo^CD64^+^ subtype showed a significantly higher percentage at 7 days after laser injury. In contrast, there were no changes in the proportion of CD8^+^ T cells after injury. All experiments were performed in triplicate. *P < 0.05 versus control of the same subtype with Dunn's multiple comparison test for post-hoc analysis.

We first investigated changes in the proportions of intraocular inflammatory cells during CNV formation following laser injury by flow cytometry. As shown in Fig 1, in WT mice, the proportion of CD4^+^ T cells recruited into the posterior segment of the eye relative to the total, increased progressively from day 3 to day 7 after laser injury. The proportions of B220^+^ B cells, granulocytes, and two intraocular macrophage subtypes (Ly6C^hi^ and Ly6C^lo^) increased significantly at day 3 after laser injury and dropped thereafter. Ly6C^int/lo^CD64^+^ subtype showed a higher percentage at day 7 after laser injury. In contrast, there were no changes in the proportion of CD8^+^ T cells after injury.

### Involvement of lymphocytes in laser-induced CNV formation

**Fig 2:**
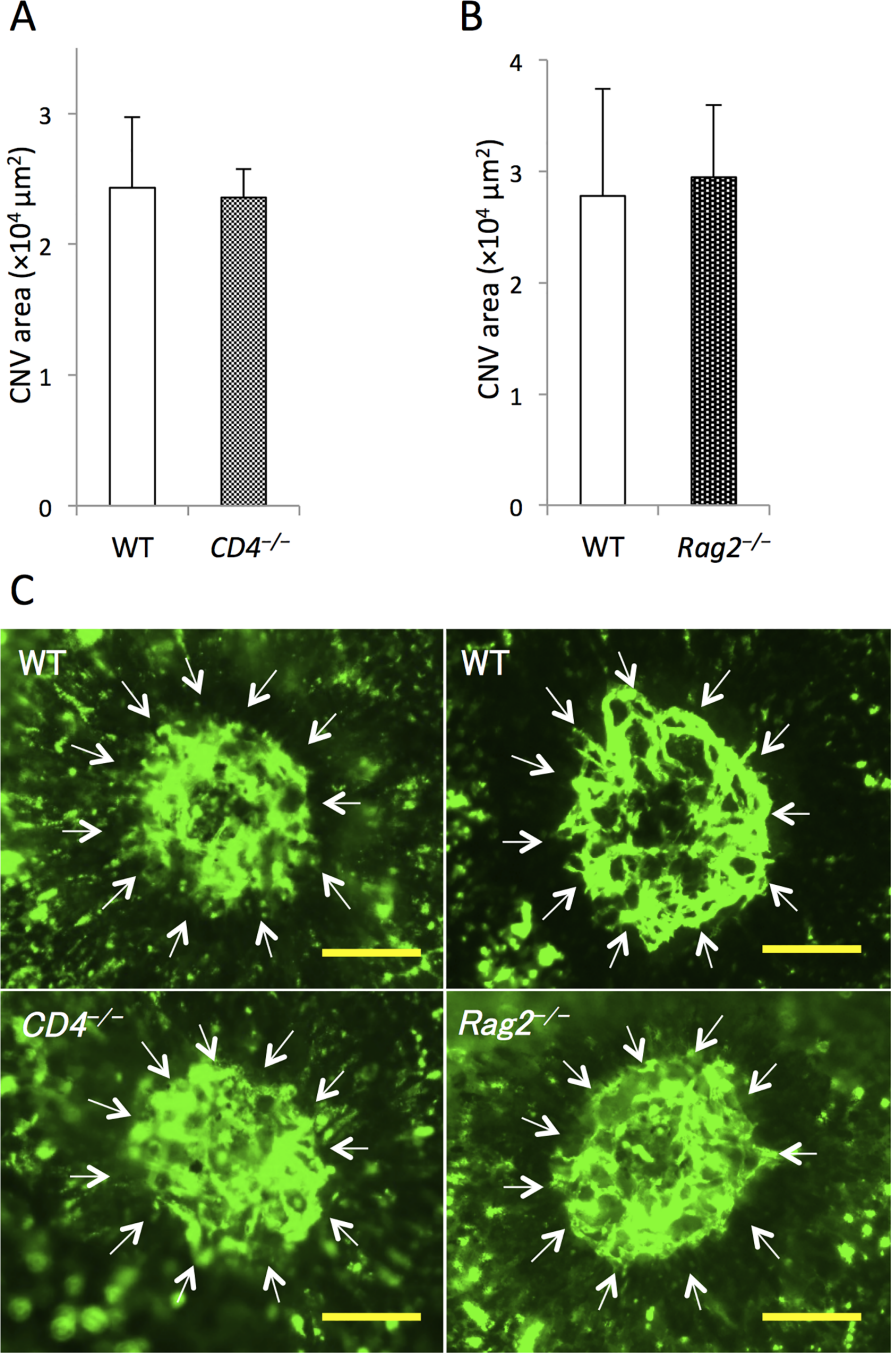
Comparison of lesion size between wild-type (WT) and transgenic mice lacking lymphocytes at day 7 after laser injury. (A) There were no differences in mean choroidal neovascularization (CNV) area between WT and *CD4*^*−/−*^ mice or (B) between WT and *Rag2*^*−/−*^ mice. (C) Representative micrographs of CNV (white arrows) in RPE-choroid flatmounts. Scale bars, 100 μm. n = 8 for all groups.

To determine the involvement of lymphocytes in CNV, next we compared CNV areas in *CD4*^*–/–*^and *Rag2*^*–/–*^mice to their respective WTs (Fig 2). There was no significant difference in lesion size between WT and *CD4*^*–/–*^mice or *Rag2*^*–/–*^mice 7 days after laser injury (Fig 2A–2C). Furthermore, we did not detect any T cells in human CNV specimens (S3 Fig).

### Contribution of macrophage subtypes to laser-induced CNV formation

**Fig 3:**
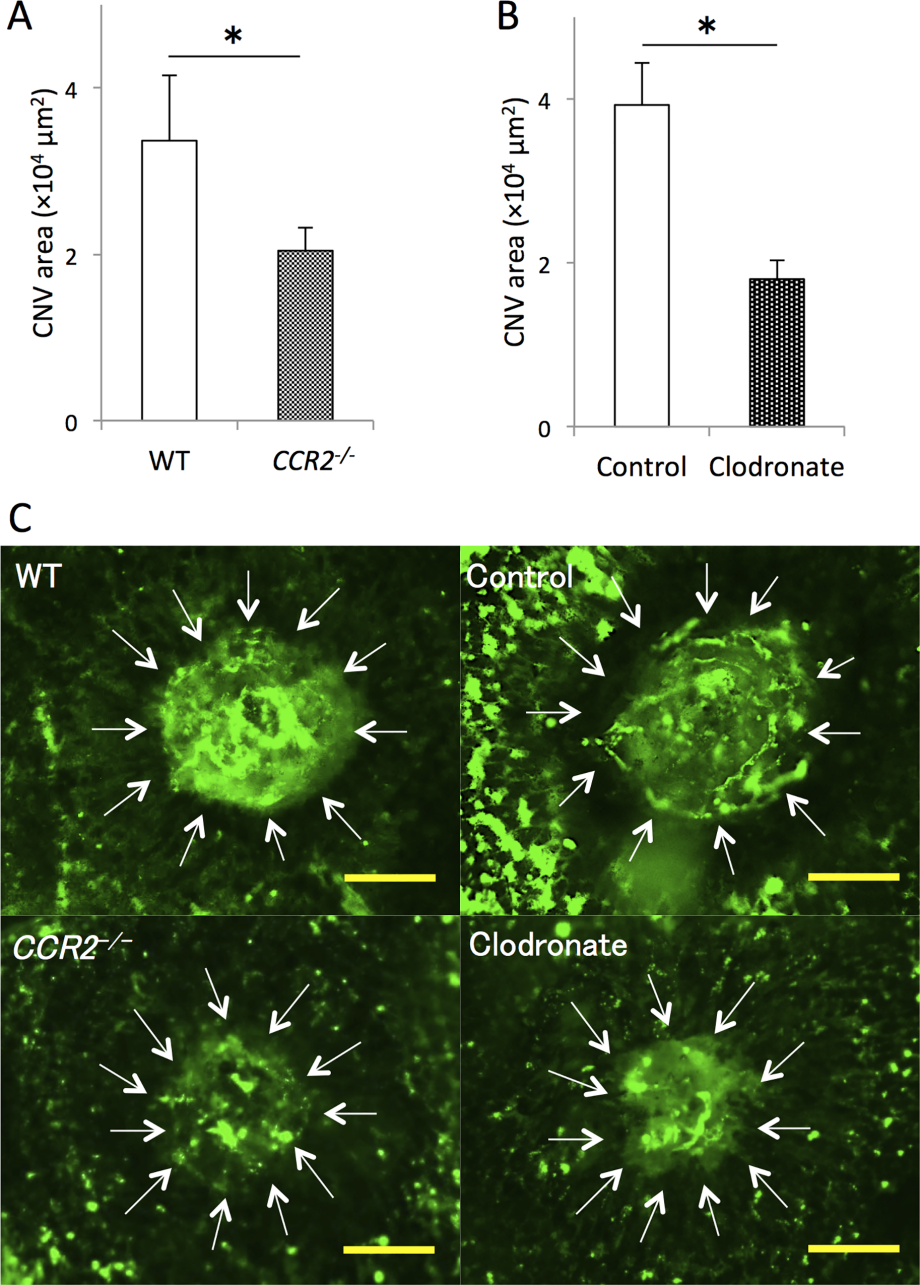
Comparison of lesion size between wild-type mice and mice lacking macrophages at the inflammatory sites or in the systemic system at day 7 after laser injury. (A) Lesion size was significantly smaller in *CCR2*^*−/−*^ mice compared with WT mice and (B) in mice injected with clodronate liposomes compared with controls. (C) Representative micrographs of CNV (white arrows) in RPE-choroid flatmounts. Scale bars, 100 μm. *P < 0.05 with Wilcoxon rank-sum test. n = 8 for all groups.

To examine the effect of monocytes/macrophages on CNV, we compared lesion size between *CCR2*^*−/−*^ mice and WT mice, and between mice in which macrophages were depleted by clodronate liposomes and controls. Lesion area was significantly reduced in *CCR2*^*−/−*^ mice (62.3% ± 6.1%) compared with WT mice 7 days after laser injury (Fig 3A and 3C). Moreover, lesion area was also significantly reduced in mice treated with clodronate liposomes (45.9% ± 5.8%) compared with controls after injury (Fig 3B and 3C).

**Fig 4:**
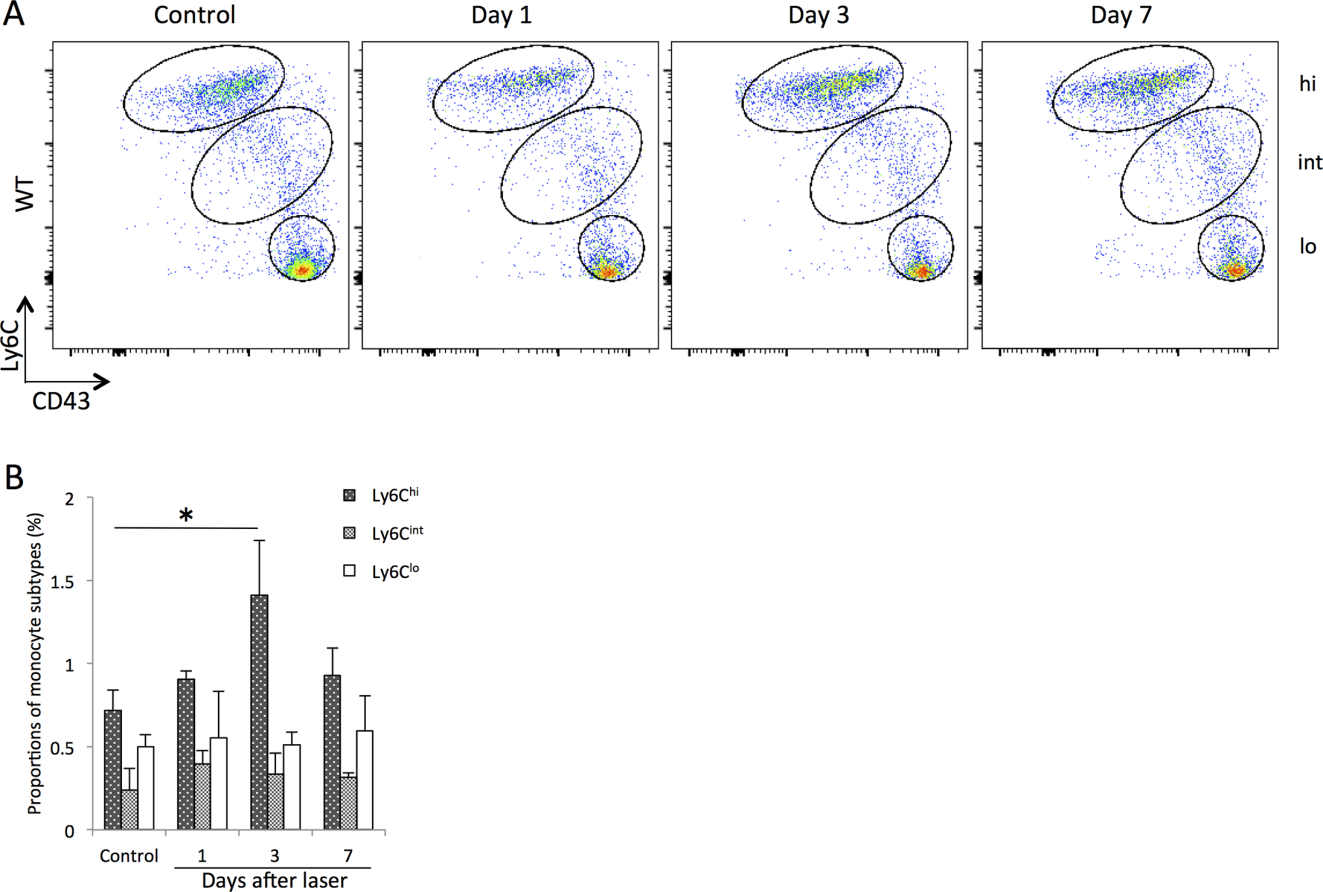
Changes in the proportions of circulating monocyte/macrophage subtypes in peripheral blood after laser injury. (A) Representative flow cytometry plots from WT mice. Monocyte subpopulations were gated based on Ly6C and CD43 expression to determine the proportions of classical (Ly6C^hi^CD43^lo^), intermediate (Ly6C^int^CD43^hi^) and nonclassical (Ly6C^lo^CD43^hi^) monocytes per total leukocytes. Hi, int, and lo correspond to Ly6C^hi^, Ly6C^int^, and Ly6C^lo^ cells, respectively. (B) The proportion of circulating Ly6C^hi^ cells were significantly higher at day 3 after laser injury, whereas there were no changes in the proportions of Ly6C^lo^ and Ly6C^int^ cells. All experiments were performed in triplicate. *P < 0.05 versus control of the same subtype with Dunn's multiple comparison test for post-hoc analysis.

Flow cytometric analysis demonstrated increased Ly6C^hi^ cells circulating in peripheral blood of WT mice at 3 days after laser injury (1.8 ± 0.4 fold compared to control). In contrast, there were no changes in the proportions of Ly6C^lo^ and Ly6C^int^ cells in peripheral blood (Fig 4A and 4B).

### Involvement of bone marrow- and spleen-derived monocytes in laser-induced CNV formation

**Fig 5:**
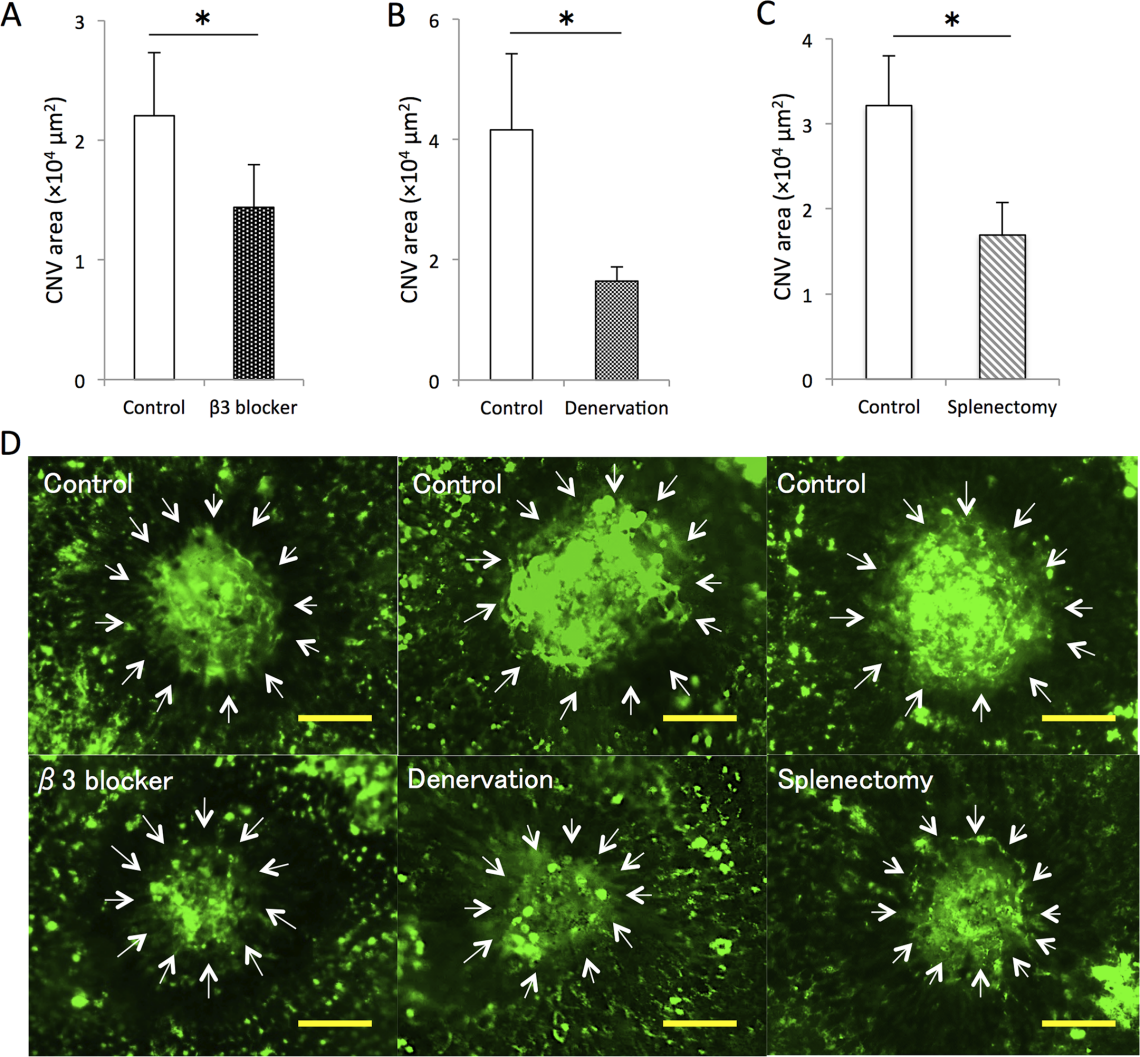
Lesion size was significantly reduced at day 7 after laser injury in mice with sympathetic nerves blockade. (A) CNV lesion size was significantly reduced in splenic-denervated mice compared with controls and (B) in mice injected with a β3 receptor antagonist injection compared with controls. (C) Splenectomized mice also showed a decreased lesion size after laser injury compared with controls. (D) Representative micrographs of CNV (white arrows) in RPE-choroid flatmounts. Scale bars, 100 μm. *P < 0.05 with Wilcoxon rank-sum test. n = 6 for all groups.

These findings indicate that monocytes/macrophages derived from bone marrow and/or the spleen, which was demonstrated to function as a reservoir for monocytes [18,20], may contribute to CNV formation. We therefore sought to confirm the involvement of bone marrow- and spleen-derived monocytes/macrophages, in exacerbating CNV lesion size in mice treated with β3 receptor antagonist and splenic-denervated or splenectomized mice. Lesion size was significantly reduced in three groups of mice (65.3% ± 1.0%, 39.5% ± 5.6%, and 52.6% ± 11.9%, respectively) compared with controls 7 days after laser injury (Fig 5A–5C).

**Fig 6:**
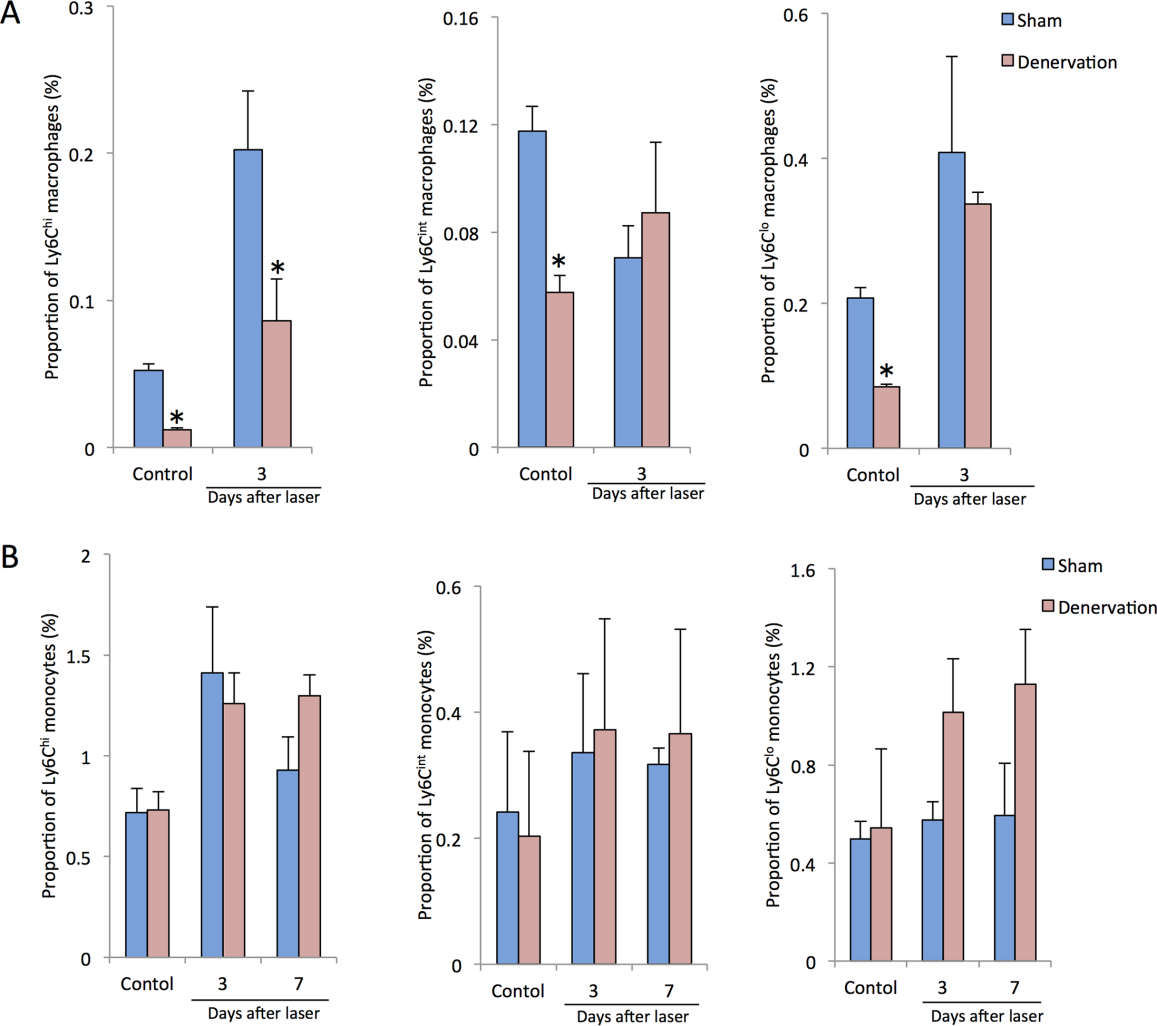
Changes in the proportions of intraocular macrophages and peripheral blood monocytes in splenic-denervated mice after laser injury. (A) Compared with controls, intraocular Ly6C^hi^ macrophages were significantly reduced by splenic denervation before laser photocoagulation and at 3 days after laser injury. Both intraocular Ly6C^int^ and Ly6C^lo^ cells showed lower proportions in splenic-denervated mice without laser treatment than those in controls, whereas there were no changes between two groups at 3 days after laser injury. (B) In contrast, there were no significant changes in the proportions of peripheral blood Ly6C^hi^, Ly6C^int^ and Ly6C^lo^ monocytes between controls and splenic-denervated mice with or without laser treatment. All experiments were performed in triplicate. *P < 0.05 versus control of the same subtype with Dunn's multiple comparison test for post-hoc analysis.

**Fig 7:**
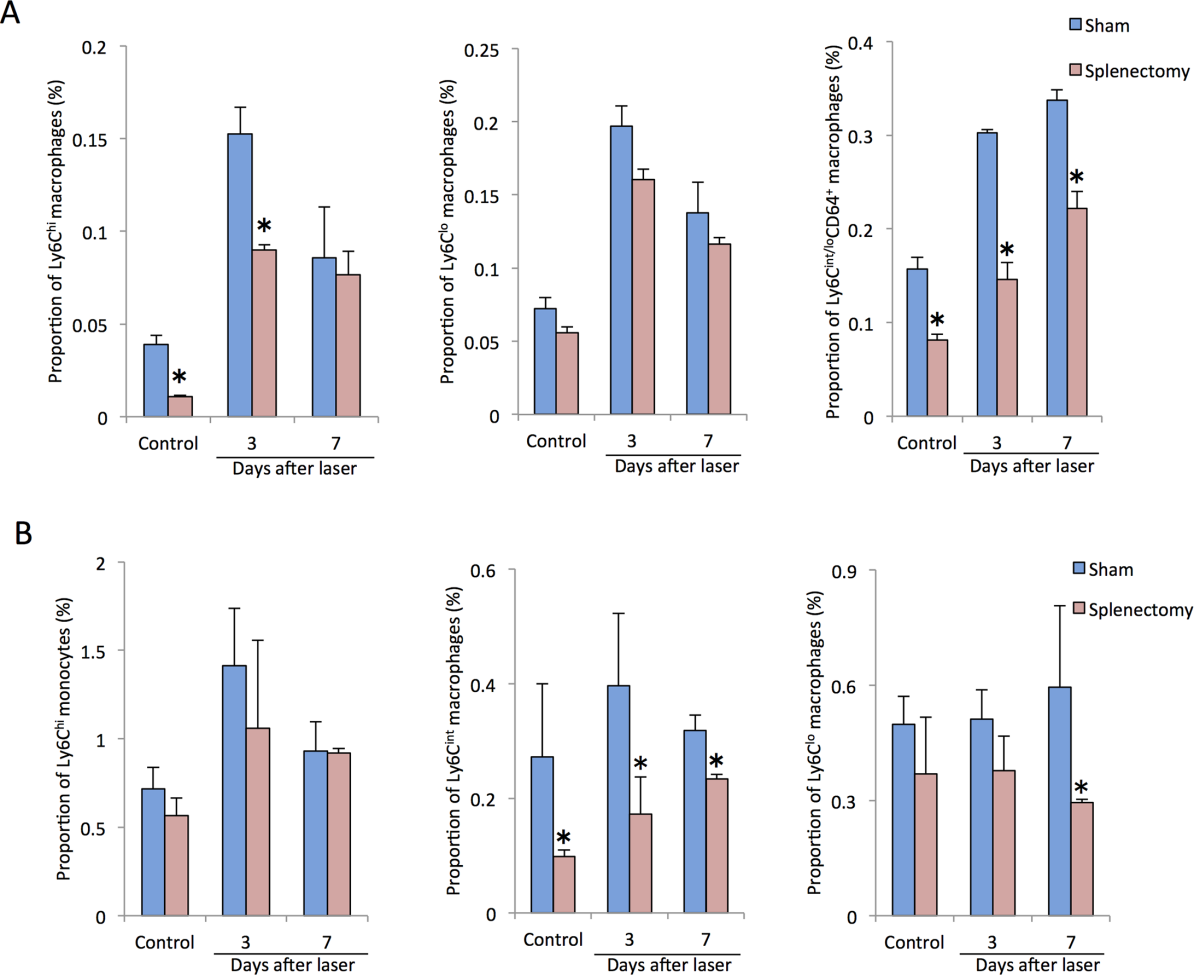
Changes in the proportions of intraocular macrophages and peripheral blood monocytes in splenectomized mice after laser injury. (A) Less Ly6C^hi^ cells were recruited into the eye in splenectomized mice than those in controls both before laser treatment and at day 3 after laser injury, and intraocular Ly6C^int/lo^CD64^+^ cells were also reduced by splenectomy from before laser photocoagulation up to 7 days after laser, whereas, there were no significant changes in Ly6C^lo^ cells between two groups with or without laser injury. (B) In the peripheral blood, a lower proportion of Ly6C^int^ cells was observed before laser treatment, at day 3 and day 7 after laser injury, and Ly6C^lo^ cells were also reduced at day 7 after laser injury. In contrast, peripheral blood Ly6C^hi^ monocytes showed no differences between controls and splenectomized mice. All experiments were performed in triplicate. *P < 0.05 versus control of the same subtype with Dunn's multiple comparison test for post-hoc analysis.

These results led us to hypothesize that spleen-derived monocytes/macrophages might play a role in the CNV formation, thus we assessed the time course of changes in the proportions of intraocular macrophage and peripheral blood monocyte in splenic-denervated and splenectomized mice after laser injury. Compared with controls, intraocular Ly6C^hi^ macrophages were significantly reduced by splenic denervation before laser photocoagulation and at 3 days after laser injury. Both intraocular Ly6C^int^ and Ly6C^lo^ cells showed lower proportions in splenic-denervated mice without laser treatment than those in controls, whereas there were no changes between two groups at 3 days after laser injury (Fig 6A). In contrast, there were no significant changes in the proportions of peripheral blood Ly6C^hi^, Ly6C^int^ and Ly6C^lo^ monocytes between controls and splenic-denervated mice with or without laser treatment (Fig 6B). Similarly, Less Ly6C^hi^ cells were recruited into the eye in splenectomized mice than those in controls both before laser treatment and at day 3 after laser injury. Additionally, intraocular Ly6C^int/lo^CD64^+^ cells were also reduced by splenectomy before laser coagulation to 7 days after laser whereas there were no significant changes in Ly6C^lo^ cells between two groups (Fig 7A). In the peripheral blood, a lower proportion of Ly6C^int^ cells was observed before laser treatment, at day 3 and day 7 after laser injury, and Ly6C^lo^ cells were also reduced at day 7 after laser injury. In contrast, peripheral blood Ly6C^hi^ monocytes showed no differences between controls and splenectomized mice (Fig 7B).

**Fig 8:**
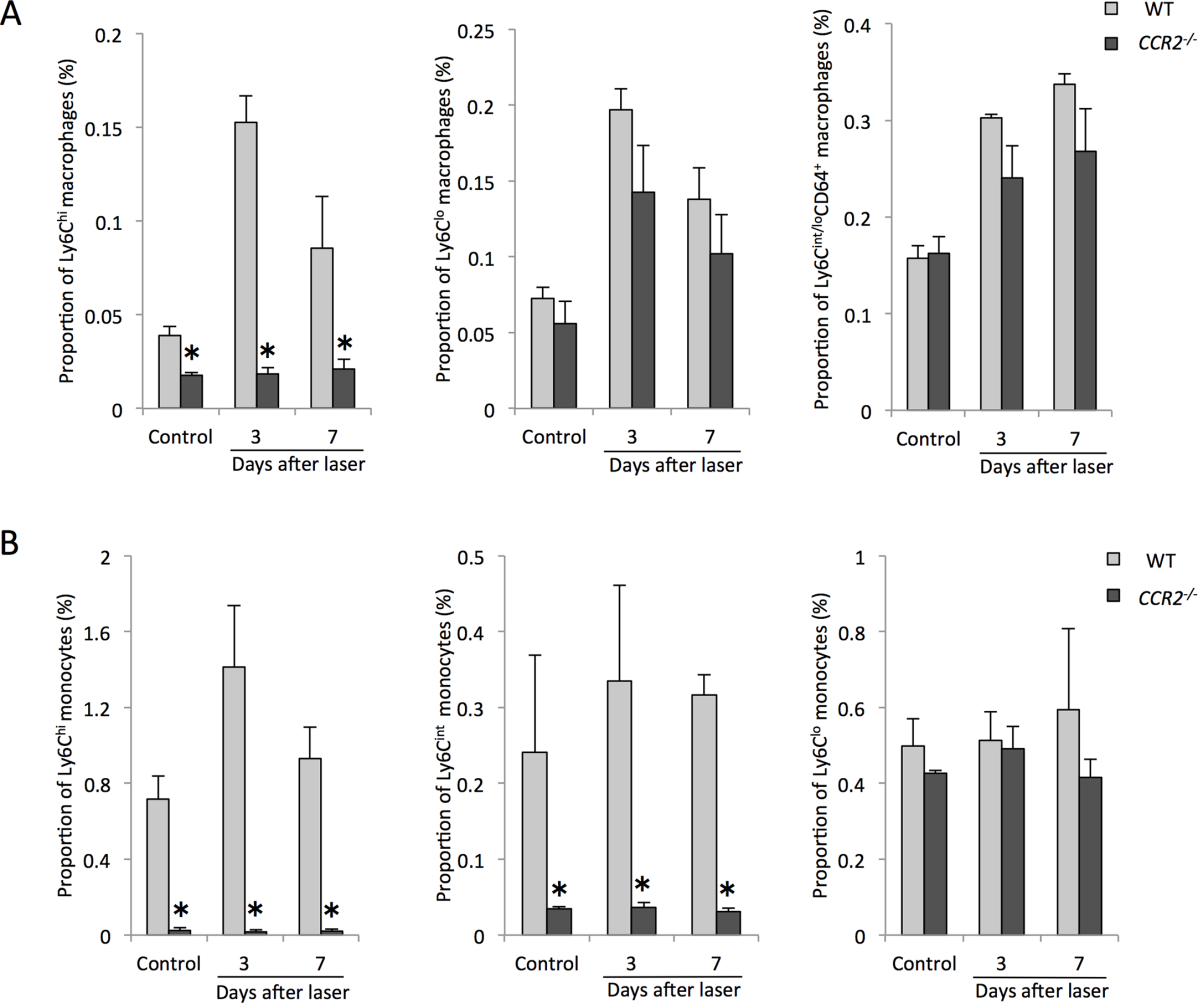
Changes in the proportions of intraocular macrophages and peripheral blood monocytes in *CCR2*^*-/-*^ mice after laser injury. (A) Ly6C^hi^ macrophage recruitment into the eye was inhibited in *CCR2*^*-/-*^ mice compared with controls without laser treatment, at 3 days and 7 days after laser injury. In contrast, there were no differences in intraocular Ly6C^lo^ and Ly6C^int/lo^CD64^+^ cells between two groups with or without laser injury. (B) The recruitment of both Ly6C^hi^ and Ly6C^int^ monocyte from bone marrow into the peripheral blood were also significantly inhibited by the absence of CCR2 expression with or without laser injury, whereas there were no significant changes in Ly6C^lo^ monocytes between two groups. All experiments were performed in triplicate. *P < 0.05 versus control of the same subtype with Dunn's multiple comparison test for post-hoc analysis.

Furthermore, to clarify the mechanism of reduced CNV size in *CCR2*^*-/-*^ mice, we also assessed the changes in intraocular macrophage and peripheral blood monocyte subtype frequencies after laser injury. Ly6C^hi^ macrophages in the eye were significantly reduced in *CCR2*^*-/-*^ mice compared with controls without laser treatment, at 3 days and 7 days after laser injury. Interestingly, there were no differences in intraocular Ly6C^lo^ and Ly6C^int/lo^CD64^+^ cells between two groups with or without laser injury (Fig 8A). Both Ly6C^hi^ and Ly6C^int^ monocytes in the peripheral blood were also reduced by the absence of CCR2 expression with or without laser injury, whereas there were no significant changes in the proportion of Ly6C^lo^ monocytes between two groups (Fig 8B).

**Fig 9:**
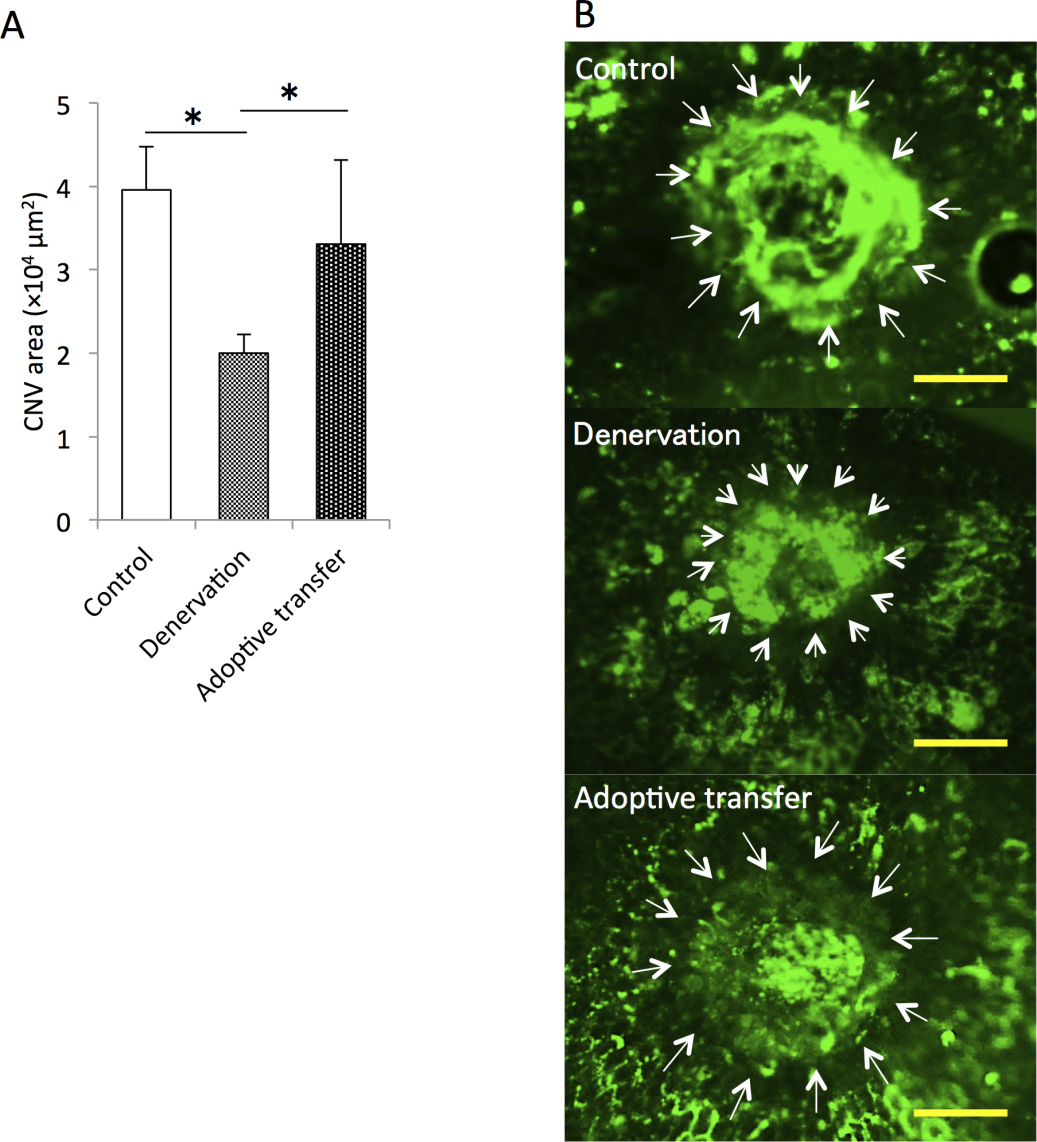
Comparison of lesion size in splenic-denervated mice with or without adoptive transfer of exogenous CD11b^+^CD115^+^Ly6C^hi^ cells, at day 7 after laser photocoagulation. (A) CD11b^+^CD115^+^Ly6C^hi^ cells harvested from the spleen were injected into splenic-denervated mice before laser photocoagulation. Lesion size, which was reduced after splenic denervation compared with controls, was significantly increased in the adoptive transfer model. (B) Representative micrographs of CNV (white arrows) in RPE-choroid flatmounts. Scale bars, 100 μm. *P < 0.05 with Wilcoxon rank-sum test. n = 6 for each group.

**Fig 10:**
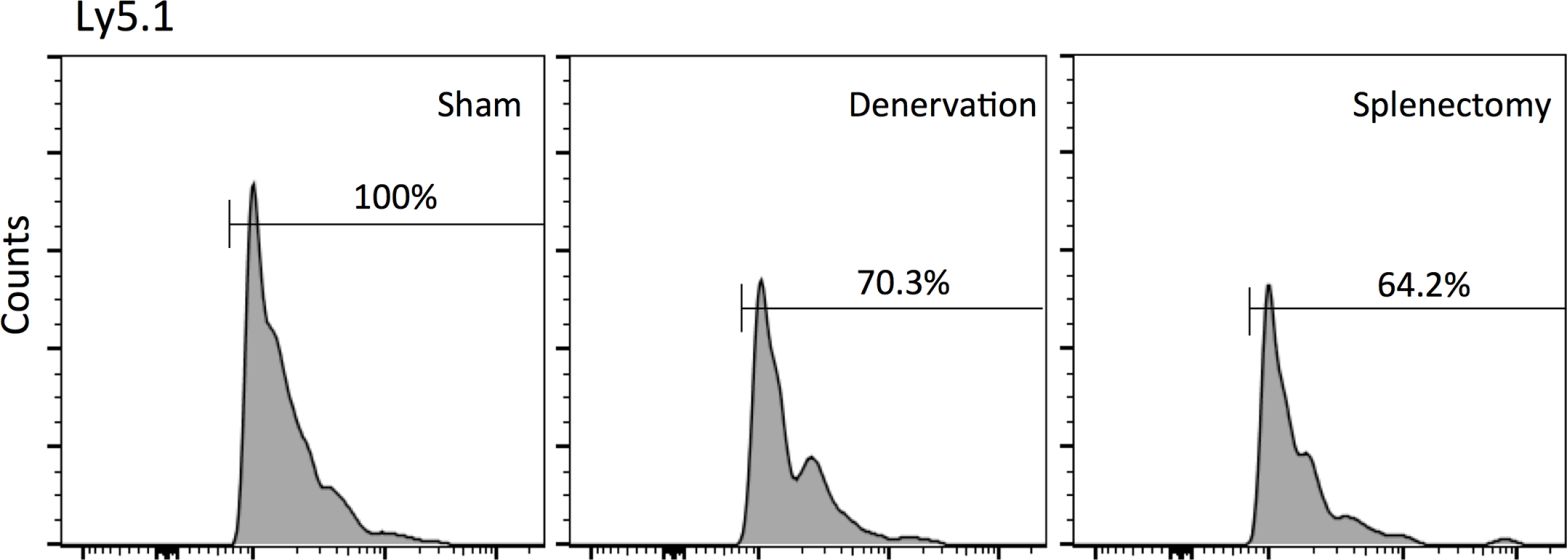
Comparison of Ly5.1^+^Ly6C^hi^ cell infiltration into the eye of Ly5.2 mice at day 3 after laser photocoagulation. We injected CD11b^+^CD115^+^Ly6C^hi^ cells harvested from the spleens of Ly5.1 mice into Ly5.2 mice before laser photocoagulation, and compared the proportion of donor-derived intraocular Ly6C^hi^ macrophages between the controls, splenic-denervated mice or splenectomized mice three days later. Compared with controls, splenic-denervated mice and splenectomized mice showed a significantly lower percentage of donor- derived Ly6C^hi^ cells in the eye after laser.

Finally, to investigate the involvement of Ly6C^hi^ macrophage/monocyte infiltration into the posterior segment of eye in CNV formation, we injected CD11b^+^CD115^+^Ly6C^hi^ cells harvested from spleen (adoptive transfer) into splenic-denervated mice before laser photocoagulation. The reduction in lesion size at 7 days after laser injury in splenic-denervated mice (51.0% ± 5.6%) compared with controls was significantly increased in the adoptive transfer model of laser-induced CNV (1.7 ± 0.5 fold of splenic-denervated values) (Fig 9A and 9B). In addition, we injected Ly6C^hi^ cells harvested from the spleens of Ly5.1 mice into Ly5.2 mice, and compared the proportion of donor-derived intraocular Ly6C^hi^ macrophages among control, splenic-denervated mice and splenectomized mice. Compared with controls, though donor-derived Ly6C^hi^ cells were infiltrated into the eyes of recipient mice, the proportion of intraocular spleen-derived Ly6C^hi^ cells was reduced in splenic-denervated and splenectomized mice at 3 days after laser injury (Fig 10).

## Discussion

Inflammatory cells such as lymphocytes and macrophages have been shown previously to play a role in the development of CNV, although their role in angiogenesis at sites of tissue injury remains controversial. In this study, we demonstrated that lymphocytes (both T cells and B cells) had no effect on laser-induced CNV formation in mice lacking CD4^+^ T cells or lacking mature T or B cells. This result is consistent with a previous report [8]. However, other groups have reported that T cells such as CD4^+^ T cells and γδT cells have a critical role in promoting pathological angiogenesis in the eye. Nussenblatt’s team showed that complement activating components, such as C5a, induced IL-17 and IL-22 production by CD4^+^ T cells from AMD patients when cocultured with monocytes and that the levels of these cytokines were also increased in the serum of AMD patients [33]. Cruz-Guillory et al reported the presence of T helper 17 (Th17) cells (effector CD4^+^ T cells), which preferentially produce IL-17, after oxidative damage induced by eye injection of the AMD-associated lipid peroxidation product carboxyethylpyrrole (CEP) and demonstrated that these cells promoted M1 polarization in vitro [7]. Moreover, IL-17 from γδT cells and innate lymphoid cells was recently found to be responsible for the increased neovascularization observed in an experimental model of CNV [34]. Together, these studies suggest that IL-17 from T cells might play a certain role in the pathogenesis and development of AMD, and the involvement of monocytes/macrophages in this process is critical. However, the role of T cells in experimental laser-induced CNV appears to be modest. Thus, it seems reasonable to suggest that the degree of lymphocyte involvement is dependent of the CNV inducer, and lymphocytes alone have little involvement in laser-induced CNV.

Some reports have shown that CCR deficiency and macrophage/monocyte depletion reduce CNV [4,5,11]. Similar results were obtained with regards to the contribution of macrophage/monocyte recruitment to the development of CNV. Moreover, our study has shown that Ly6C^hi^ cell infiltration into the peripheral blood and the eye was inhibited in *CCR2*^*-/-*^ mice. This is consistent with previous reports indicating that CCR2 is critical for the egress of Ly6C^hi^ monocytes from bone marrow and infiltration into inflamed tissue [35,36]. In contrast, peripheral blood and intraocular Ly6C^lo^ cells did not show changes compared with WT with or without laser treatment. Ly6C^lo^ monocytes/macrophages represent the noninflammatory subtype. They do not express CCR2 but high levels of CXCR1 and give rise to resident macrophages in noninflamed tissues [37]. CX3CL1/CX3CR1 signaling has been demonstrated to be required for the accumulation of Ly6C^lo^ monocytes/macrophages [17,37]. Taken together, our results support the idea that CCR2 deficiency does not change distribution of the nonclassical monocytes [9]. Our findings have also suggested that Ly6C^hi^ macrophages/monocytes are associated with exacerbation of experimental CNV. In contrast with our data, another group has demonstrated that macrophages showed an anti-angiogenic function and inhibits the growth of abnormal blood vessels in the eye. Apte and colleagues reported that GM-CSF- or IFN-γ-cultured macrophages inhibited CNV upon injection into the eyes of host mice (1 × 10^5^ cells/eye) at the time of tissue injury [6,38]. Although it is likely that some macrophages may protect against CNV, the phenotype of the cells was not sufficiently characterized in these studies. Especially, since there was no documentation about the *Rd8* mutation in the mice used in these previous studies [6,38], the results should be interpreted with caution. In contrast, in the present study, we used an adoptive transfer model, as the procedure is useful for studying the effects of immune cells on tissues in the absence of other immune cells, and it has been widely used in studies of tumors, chronic kidney disease, and cardiovascular disease [39–41]. Unlike in previous experiments where an excessive number of macrophages were injected into eyes, our models are more suitable for describing the influence of macrophages in common physiological and pathophysiological conditions. Since there are many conversions among macrophage subtypes, it seems to be difficult to classify macrophage/monocyte subpopulation clearly. Whether our findings depend on tissue specificity or not, the role of each subtype of macrophage in angiogenesis in the eye remains unclear and further study is still required.

Sympathetic nervous system stimulation associated with stress has been shown to promote angiogenesis in a mouse model of ovarian carcinoma [42]. In our study, sympathetic blockade by β3 blocker injection or splenic denervation markedly suppressed the recruitment of macrophages and inhibited experimental CNV. In contrast, other groups reported that loss of sympathetic innervation to the choroid by cervical sympathectomy resulted in a significant increase in the numbers of venules and sizes of arterioles at 6 weeks post surgery in rats [21,43]. Although the inflammatory response is essential in both experimental CNV models and AMD patients, the slow process of neovascularization in AMD patients is undoubtedly more complex than that following photocoagulation injury. Nonetheless, our data suggest that sympathetic nerve stimulation might be a critical factor leading to the early exacerbation of CNV.

In a mouse model of myocardial infarction, when seeking the source of surplus monocytes in plaque, hematopoietic stem and progenitor cells have been demonstrated to be liberated from bone marrow via sympathetic nervous system signaling after myocardial infarction or stroke. The progenitors then seed the spleen yielding a sustained boost in monocyte and macrophage production. Increased monocyte/macrophage recruitment has been showed to aggravate atherosclerosis and promote recurrent myocardial ischemia [32]. It is likely that a similar series of pathophysiologic events may occur in the eye. Since the pathogenesis of AMD is likely to be multifactorial, involving a complex interaction of metabolic, genetic, and environmental factors [44]; it was presumed that the early pathogenic events of AMD such as oxidative stress and subretinal ischemia, could increase sympathetic nervous system activity, leading to the release of monocytes both from bone marrow and spleen; then with aggravation of inflammation, macrophages could be released into the peripheral blood, resulting in increased intraocular macrophages and exacerbation of neovascularization. To verify this hypothesis, we assessed the changes of intraocular and peripheral blood monocytes/macrophages during the process of CNV formation in splenic-denervated and splenectomized mice. However, there were no significant changes in the proportions of all three peripheral blood monocyte subtypes between controls and splenic-denervated mice with or without laser treatment. Although a lower proportion of Ly6C^int^ cells was observed in splenectomized mice with or without laser injury, and Ly6C^lo^ cells were reduced at day 7 after laser injury; peripheral blood Ly6C^hi^ monocytes showed no differences between controls and splenectomized mice. Our findings suggested that increased Ly6C^hi^ monocytes in the peripheral blood were mostly derived from the bone marrow in laser-induced mouse model of CNV, and even when splenic denervation or splenectomy was performed, they still egressed from bone marrow into peripheral blood. In addition, decreased Ly6C^int/lo^CD64^+^ cells recruitment into the eye by splenectomy may be due to the reduction of peripheral blood Ly6C^int^ and Ly6C^lo^ monocytes. However, not only was CNV lesion size reduced by splenic denervation or splenectomy, Ly6C^hi^ macrophage recruitment into the eye was also suppressed in these two mouse models. Lesion size in splenic-denervated mice was increased through adoptive transfer of spleen-derived Ly6C^hi^ cells, suggesting spleen-derived Ly6C^hi^ cells might play a role in the development of CNV even though they were not directly recruited into the peripheral blood. These findings sharply contrast to previous studies in the myocardial infarction model, in which it has been suggested that progenitor cells released from bone marrow niches, entered into the spleen to yield a sustained boost in monocyte/macrophage production, resulting in increased monocytopoiesis and plaque rupture [32]. We assumed that Ly6C^hi^ macrophages could be increased via adoptive transfer in the eyes of spleen-derived or splenectomized mice; however, donor-derived Ly6C^hi^ cell infiltration into the eye was unexpectedly reduced both in splenic-denervated and splenectomized mice compared with controls. These findings indicate that the spleen is not the main source for circulating peripheral blood Ly6C^hi^ monocytes after laser injury in experimental CNV model; alternatively, suggest that signaling through sympathetic nervous stimulation and/or the Ly6C^hi^ cells from the spleen may be involved in monocyte migration/recruitment into the eye, especially Ly6C^hi^ cells. Though our findings may depend on tissue specificity and animal species, the involvement of sympathetic nervous situation in CNV is complex and remains unclear; thus, further studies examining the involvement of the automatic nervous system in intraocular CNV are warranted.

Splenectomy is not a realistic treatment, but treatment with systemic β blockers to decrease local inflammation might be feasible. A recent clinical study investigating the effects of systemic β blockers found no benefit for prevention of wet AMD [45]. In that study, however, hypertension might have been a confounding factor. Another study reported that systemic β blocker treatment may have reduced the need for repeated intravitreal injections of bevacizumab in patients with CNV [46], indicating that systemic β blocker treatment may reduce the number of intravitreal injections of anti-vascular endothelial growth factor agents. Although there is no strong evidence to suggest that systemic β blocker treatment prevents CNV, it may prove to be a useful adjunct treatment for AMD by reducing bone marrow-derived macrophage infiltration. In addition, macrophages have been demonstrated to play an important role in the formation of fibrosis, which also causes vision loss in AMD [47,48]. Inhibition of macrophage/monocyte recruitment by sympathetic blockade might contribute to a reduction in subretinal fibrosis. Further studies are needed to clarify the effects of β3 receptor antagonism on splenic monocyte egression in the current CNV model.

In this study, we focused on the link between macrophage recruitment and CNV. Moreover, plasticity and polarization are hallmarks of cells of monocyte and macrophage. Depending on the tissue context and in response to various signals, macrophages can be classically (M1) activated in the presence of IFN-γ and TLR ligands or alternatively (M2) activated in the presence IL-4/IL-13. M1 phenotype is characterized by antibacterial and proinflammatory functions. In contrast, M2 phenotype is considered to be involved in promotion of tissue remodeling and tumor progression [49]. The plasticity and polarization of macrophages play a critical role in inflammation and angiogenesis. Targeting polarized activation of macrophages will be an important issue in future research.

Our data show that sympathetic blockade inhibits CNV formation via reduction of macrophage/monocyte recruitment into the eye. Systemic β blockers may be an effective therapy for exudative AMD.

## Supporting Information

S1 Fig Gating strategy for peripheral blood monocytes. We identified the peripheral blood monocytes by two gating strategies. (A) Monocytes were identified as CD45^+^CD11b^+^Ly6G^-^CD115^+^. Monocyte subpopulations were gated based on Ly6C and CD43 expression to determine the proportions of classical (Ly6C^hi^CD43^lo^), intermediate (Ly6C^int^CD43^hi^) and nonclassical (Ly6C^lo^CD43^hi^) monocytes per total leukocytes. (B) The proportion of circulating Ly6C^hi^ cells was significantly higher at day 3 after laser injury compared with control, whereas there were no changes in the proportions of Ly6C^lo^ and Ly6C^int^ cells. (C) Monocytes were gated by another antibody combination and identified as CD11b^+^Ly6G^-^F4/80^+^. Monocyte subpopulations were gated based on Ly6C and CD11b expression to determine the proportion of: classical (Ly6C^hi^CD11b^+^), intermediate (Ly6C^int^CD11b^+^) and nonclassical (Ly6C^lo^CD11b^+^) monocytes per total leukocytes. (D) Similar results were showed with Ly6C and CD43 gating strategy. Compared with control, the proportion of Ly6C^hi^ cells was significantly increased at day 3 after laser injury, and there were no changes in the proportions of Ly6C^lo^ and Ly6C^int^ cells. All experiments were performed in triplicate. *P < 0.05 versus control of the same subtype with Dunn's multiple comparison test for post-hoc analysis.
(TIFF)

S2 Fig Gating strategy for intraocular macrophages. We identified intraocular macrophages by two gating strategies. (A) Macrophages were identified as CD45^+^CD11b^+^Ly6G^-^. Macrophage subpopulations were gated based on Ly6C and CD64 expression to determine the proportions of Ly6C^hi^, Ly6C^lo^, and Ly6C^int/lo^CD64^+^ subtype per total retinal and RPE/choroidal cells. (B) Compared with control, the proportions of intraocular Ly6C^hi^ and Ly6C^lo^ cells were significantly higher at day 3 after laser injury, whereas Ly6C^int/lo^CD64^+^ cells showed a higher percentage at 7 days after laser injury. (C) Monocytes were gated by another antibody combination and identified as CD11b^+^Ly6G^-^F4/80^+^. Macrophage subpopulations were gated based on Ly6C and CD11b expression to determine the proportions of Ly6C^hi^, Ly6C^lo^, and Ly6C^int^ subtype per total retinal and RPE/choroidal cells. (D) Compared with control, the proportions of Ly6C^hi^ and Ly6C^lo^ cells were significantly increased at 3 days and 7 days after laser injury, respectively. However, there were no changes in the proportion of Ly6C^int^ cells, suggesting that Ly6C^int/lo^CD64^+^ cells showed a similar tendency with Ly6C^lo^ subpopulation while they were different from Ly6C^int^ subpopulation. All experiments were performed in triplicate. *P < 0.05 versus control of the same subtype with Dunn's multiple comparison test for post-hoc analysis.
(TIFF)

S3 Fig Immunostaining of human CNV specimens. The infiltration of T cells into the human CNV lesion was evaluated by immunostaining. The specimens of human CNV were kindly provided by Dr Hiroyuki Nakashizuka (Division of Ophthalmology, Department of Visual Science, Nihon University School of Medicine, Tokyo, Japan). The specimens were immunostained with anti-human CD3 antibody (BioLegend). Sections of hematoxylin and eosin (HE) staining, control, and CD3 staining are shown. No T cells were detected in human CNV (black arrows). Scale bars, 200 μm.
(TIFF)
