# Supplementary figures and images for: Choroidal Neovascularization Is Inhibited in Splenic-Denervated or Splenectomized Mice with a Concomitant Decrease in Intraocular Macrophage
Source: PLoS One. 2016 Aug 17;11(8):e0160985. doi: 10.1371/journal.pone.0160985 (PMC4988653; doi:10.1371/journal.pone.0160985)

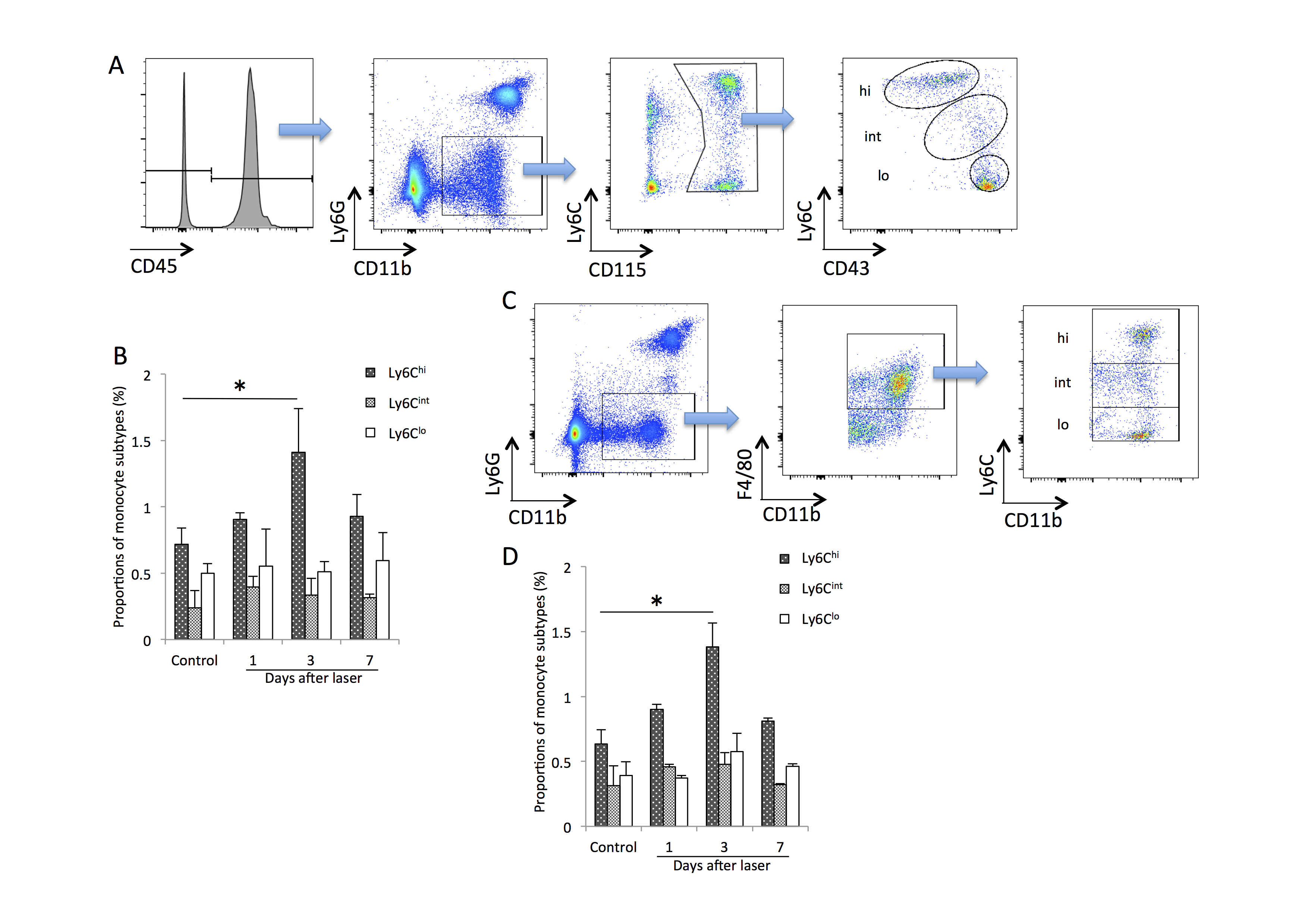

Supplement: S1 Fig — We identified the peripheral blood monocytes by two gating strategies. (A) Monocytes were identified as CD45+CD11b+Ly6G-CD115+. Monocyte subpopulations were gated based on Ly6C and CD43 expression to determine the proportions of classical (Ly6ChiCD43lo), intermediate (Ly6CintCD43hi) and nonclassical (Ly6CloCD43hi) monocytes per total leukocytes. (B) The proportion of circulating Ly6Chi cells was significantly higher at day 3 after laser injury compared with control, whereas there were no changes in the proportions of Ly6Clo and Ly6Cint cells. (C) Monocytes were gated by another antibody combination and identified as CD11b+Ly6G-F4/80+. Monocyte subpopulations were gated based on Ly6C and CD11b expression to determine the proportion of: classical (Ly6ChiCD11b+), intermediate (Ly6CintCD11b+) and nonclassical (Ly6CloCD11b+) monocytes per total leukocytes. (D) Similar results were showed with Ly6C and CD43 gating strategy. Compared with control, the proportion of Ly6Chi cells was significantly increased at day 3 after laser injury, and there were no changes in the proportions of Ly6Clo and Ly6Cint cells. All experiments were performed in triplicate. *P < 0.05 versus control of the same subtype with Dunn's multiple comparison test for post-hoc analysis. (TIFF) [file pone.0160985.s001.tiff]

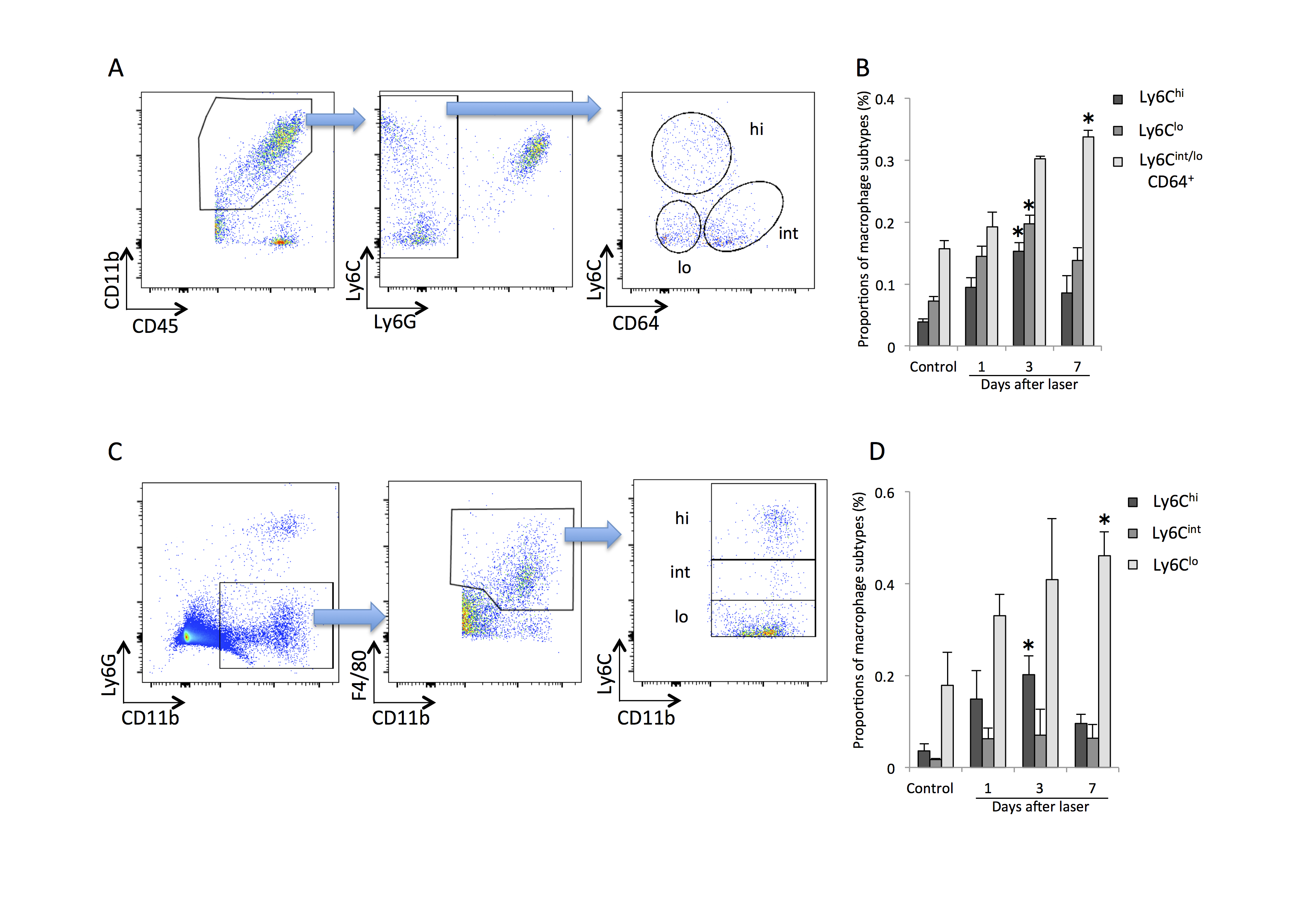

Supplement: S2 Fig — We identified intraocular macrophages by two gating strategies. (A) Macrophages were identified as CD45+CD11b+Ly6G-. Macrophage subpopulations were gated based on Ly6C and CD64 expression to determine the proportions of Ly6Chi, Ly6Clo, and Ly6Cint/loCD64+ subtype per total retinal and RPE/choroidal cells. (B) Compared with control, the proportions of intraocular Ly6Chi and Ly6Clo cells were significantly higher at day 3 after laser injury, whereas Ly6Cint/loCD64+ cells showed a higher percentage at 7 days after laser injury. (C) Monocytes were gated by another antibody combination and identified as CD11b+Ly6G-F4/80+. Macrophage subpopulations were gated based on Ly6C and CD11b expression to determine the proportions of Ly6Chi, Ly6Clo, and Ly6Cint subtype per total retinal and RPE/choroidal cells. (D) Compared with control, the proportions of Ly6Chi and Ly6Clo cells were significantly increased at 3 days and 7 days after laser injury, respectively. However, there were no changes in the proportion of Ly6Cint cells, suggesting that Ly6Cint/loCD64+ cells showed a similar tendency with Ly6Clo subpopulation while they were different from Ly6Cint subpopulation. All experiments were performed in triplicate. *P < 0.05 versus control of the same subtype with Dunn's multiple comparison test for post-hoc analysis. (TIFF) [file pone.0160985.s002.tiff]

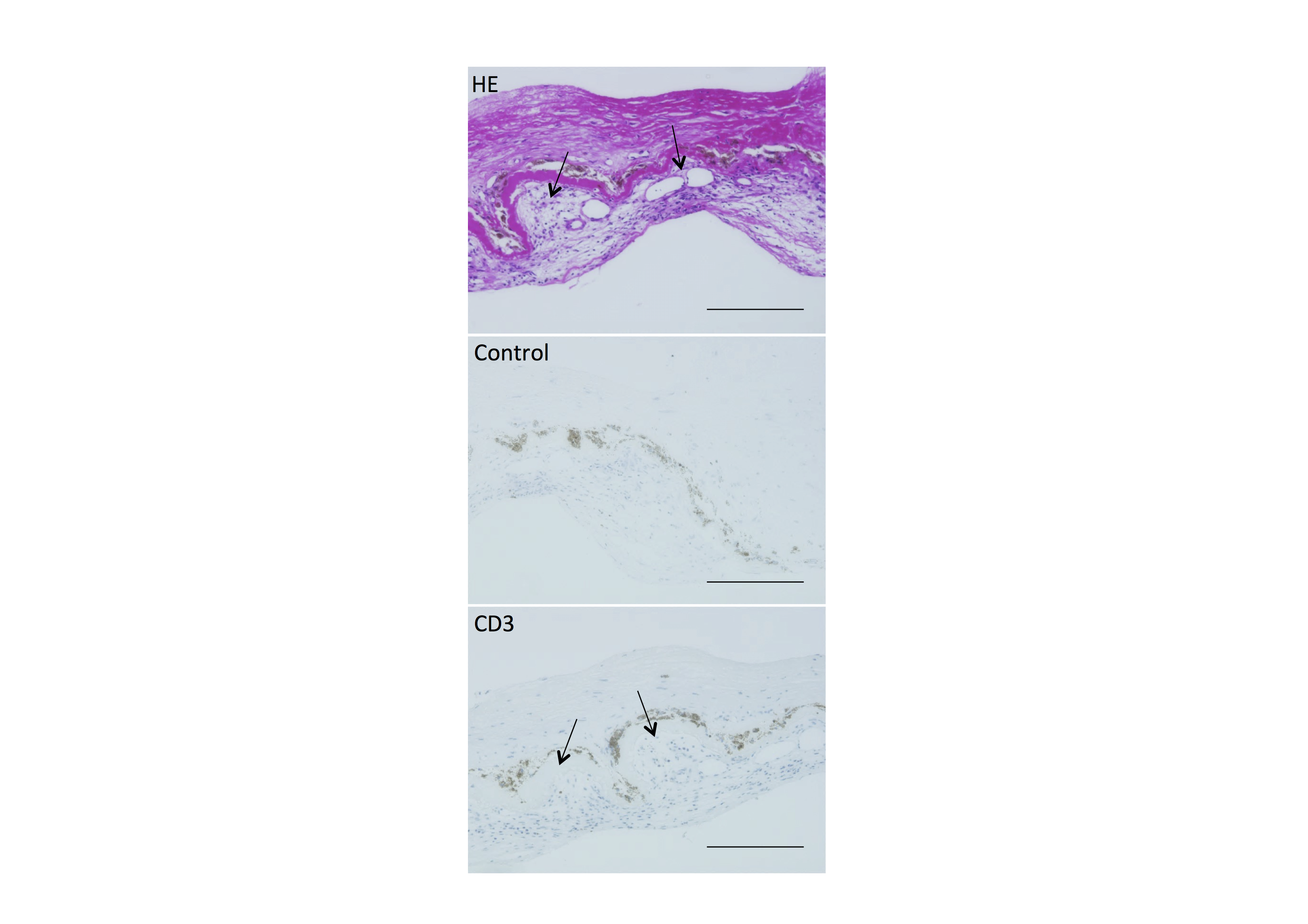

Supplement: S3 Fig — The infiltration of T cells into the human CNV lesion was evaluated by immunostaining. The specimens of human CNV were kindly provided by Dr Hiroyuki Nakashizuka (Division of Ophthalmology, Department of Visual Science, Nihon University School of Medicine, Tokyo, Japan). The specimens were immunostained with anti-human CD3 antibody (BioLegend). Sections of hematoxylin and eosin (HE) staining, control, and CD3 staining are shown. No T cells were detected in human CNV (black arrows). Scale bars, 200 μm. (TIFF) [file pone.0160985.s003.tiff]
